# Abscisic Acid, Stress, and Ripening (*Tt*ASR1) Gene as a Functional Marker for Salt Tolerance in Durum Wheat

**DOI:** 10.1155/2020/7876357

**Published:** 2020-01-31

**Authors:** Karama Hamdi, Faiçal Brini, Najla Kharrat, Khaled Masmoudi, Inès Yakoubi

**Affiliations:** ^1^Biotechnology and Plant Improvement Laboratory, Centre of Biotechnology of Sfax (CBS), University of Sfax, B.P “1177” 3018, Sfax, Tunisia; ^2^Laboratory of Molecular and Cellular Screening Process, Centre of Biotechnology of Sfax (CBS), University of Sfax, B.P “1177” 3018, Sfax, Tunisia; ^3^College of Food and Agriculture, Arid Land Department, United Arab Emirates University, Al Ain, UAE

## Abstract

In semiarid Mediterranean agroecosystems, drought and salinity are the main abiotic stresses hampering wheat productivity and yield instability. Abscisic acid, stress, and ripening (ASR) are small plant proteins and play important roles in different biological processes. In the present study, the *Tt*ASR1 gene was isolated and characterized for the first time from durum wheat (*Tritucum turgidum* L. subsp. *durum*). *Tt*ASR1 is a small gene, about 684 bp long, located on chromosome 4AL, encoding a protein of 136 amino acid residues consisting of a histidine-rich N terminus and C-terminal conserved ABA-WDS domain (Pfam PF02496). Our results showed that *Tt*ASR1 protein could function as a chaperone-like protein and improve the viability of *E. coli* under heat and cold stress and increase the *Saccharomyces cerevisiae* tolerance under salt and osmotic stress. Transcript expression patterns of *Tt*ASR1 revealed that ASRs play important roles in abiotic stress responses in diverse organs. Indeed, *Tt*ASR1 was upregulated in leaves by different developmental (ABA) and environmental signals (PEG, salt). In *cv*. Mahmoudi (salt-tolerant Tunisian durum landraces) roots, *Tt*ASR1 was upregulated by salt stress, while it was downregulated in cv. Azizi (salt-sensitive Tunisian durum landraces), supporting the implication of this gene in the salt tolerance mechanism. Taken together and after validation in the plant system, the *Tt*ASR1 gene may provide a potential functional marker for marker-assisted selection in a durum wheat breeding program for salt tolerance.

## 1. Introduction

Abiotic stresses such as extreme temperature, drought, salinity, heavy metals, and radiation represent the most limiting factors for agricultural productivity worldwide [1]. To cope with such challenges, plants developed some adaptation strategies to escape the changing environmental conditions. However, abiotic stress responses in plants are complexes due to the interrelationship of mechanisms and variability of involved molecules. Various stress signals share with abscisic acid (ABA) common elements in the signaling pathway, and to maintain cellular homeostasis, these elements cross-talk with each other. In fact, the phytohormone, ABA, is one among many endogenous messengers implicated in the regulation of the plant's water status and controlled their adaptive process [2]. It has been shown that ABA signaling could be involved in regulating components [3, 4]. It could be also implicated in the regulation of many processes in plants; these include specific expression patterns during seed development, drought, cold, and salt responses [5].

Abscisic acid, stress, and ripening (ASR) gene was demonstrated to be regulated by ABA and abiotic stresses or during fruit ripening [6]. Back in 1993, the first ASR gene has been identified in tomato from the cDNA library induced by ABA [7]. Subsequently, other studies demonstrated that the ASR gene was induced by ABA in a range of plant species [8].

Over the past 20 years, ASR genes have been reported in many plant species ranging from gymnosperms to monocots and dicot [9]. The analysis of their expression pattern shows that these genes are present in a variety of tissues. In tomato, ASR1 transcript was found in leaves and fruit pericarp [7] and ASR2 in roots [10]. *Pinus* ASR (lp3) was expressed mostly in roots under water-deficit conditions [11]. Consistently, ASR proteins were present, in lily pollen, mainly during the drying stage and developing pollen [9]. ASR genes belong to a small gene family with a very simple structure: two exons separated by an intron [12]. Battaglia et al. have proposed ASR as a group of late embryogenesis abundant (LEA) proteins [13]. These proteins (LEA) are widespread in land plants. Most of them belong to the “hydrophilins” family, a group of highly hydrophilic, intrinsically unstructured proteins (IUPs) characterized by a biased amino acid composition enriched in gly and other small residues that favor a flexible conformation [14]. Surprisingly, these genes are not present in Arabidopsis [6] and yeast cells [9].

In our previous study, we showed that TtASR1 protein is intrinsically disordered proteins (IDP) and undergoes structural transitions under dehydration, heat, and desiccation using both biochemical and biophysical methods [15]. To validate further the candidate gene TtASR1 for abiotic stress tolerance and gain insight into its function, the TtASR1 was first isolated and characterized for the first time from durum wheat (*Tritucum turgidum* L. subsp. *durum*). TtASR1 expression pattern gene under NaCl, polyethylene glycol (PEG), and ABA, in leaves and roots of two Tunisian durum wheat landraces, displaying contrasting tolerance to salt stress, *cv.* Mahmoudi and *cv.* Azizi, was carried out. Furthermore, the biological function of the TtASR1 gene was analyzed by the overexpression in *E. coli* and the yeast *Saccharomyces cerevisiae.*

## 2. Materials and Methods

### 2.1. Plant Materials and Stress Treatments

Two local Tunisian genotypes of tetraploid *T. turgidum* L. subsp. *durum* (2*n* = 4*x* = 28), with contrasting tolerance to salinity, *cv.* Mahmoudi (salt-tolerant) and *cv.* Azizi (salt susceptible), were used for ASR expression profile analysis. Mahmoudi was used for ASR gene cloning; seeds were provided from the Kef Higher Agricultural School-Tunisia. All seeds were initially surface sterilized by a 0.5% NaClO wash for 15 min, rinsed three times with sterile water, and germinated on wet Whatman paper filter placed in Petri dishes after 2 days in the dark. Ten-day-old seedlings grown were subjected to stress. For salinity and drought treatments, seedlings were incubated in 200 mM NaCl or 15% polyethylene glycol 6000 (PEG 6000). For signaling molecule treatments, seedlings were incubated in 100 *μ*M ABA. Both control and stressed seedlings were sampled at 24 h of treatments. Leaves and roots were harvested and frozen in liquid nitrogen for RNA isolation.

### 2.2. Durum Wheat ASR Primers Design and Amplification of Its Genomic Sequence

A set of full ASR protein sequences belonging to monocot plant species were retrieved from Genebank of NCBI (http://www.ncbi.nlm.nih.gov/protein/) using the ABA_WDS consensus domain as a query. For the ASR proteins, showing an interesting protein sequence homology (rice (N° AAB96681.1), maize (N° CAD12677.1), lily (N° AAF15307.1), banana (N° AAT35818.1), and sugarcane (N° AAT57940.1)), the corresponding nucleotide sequences were retrieved and the alignment was performed using MUSCUL. The conserved regions, identified on nucleotide sequence alignment, were used to design P1 and P2 primers (Supplementary [Supplementary-material supplementary-material-1]). PCR was performed on genomic DNA extracted from the Mahmoudi and Azizi genotypes. PCR product (approximately 500 pb) was purified from agarose gel, cloned in pGEM-T easy vector, and sequenced using ABI PRISM automated sequencer.

The obtained sequence was blasted against Graingene wheat transcriptome database (https://wheat.pw.usda.gov/GG2/WheatTranscriptome/). The contig (UCW_Tt-k45_contig_942), having a null *E* value, was used to design a second set of primers (Supplementary [Supplementary-material supplementary-material-1]) for ASR gene isolation. A second PCR was performed on genomic DNA extracted from Mahmoudi and the PCR product (700 pb) was purified from agarose gel, cloned in pGEM-T easy vector, and sequenced using ABI PRISM automated sequencer. The obtained sequence was analyzed by BLAST (https://blast.ncbi.nlm.nih.gov/Blast) and conserved database domain CDD (http://www.ncbi.nlm.nih.gov/Structure/cdd). The open reading frame and the structure of TtASR1 gene were made using Softberry (http://www.softberry.com/cgi-bin/programs/gfind/fgenesh.pl).

### 2.3. RNA Extraction and Semiquantitative RT-PCR

Total RNA was isolated from approximately 200 mg of durum wheat leaves and roots according to the Trizol method (Invitrogen) and following the manufacturer's instructions. RNA was treated with RNase-free DNase to remove any contaminating DNA. Reverse transcription reactions were performed for 1 h at 37°C using MML-reverse transcriptase (Invitrogen) and oligo-dT. First-strand complementary DNA (cDNA) was used as a template for PCR amplifications using a couple of specific primers designed from the obtained ASR sequence. Control amplifications in the absence of the reverse transcriptase were also performed to rule out any amplification caused by the presence of contaminating DNA. Expression levels of target genes were normalized using actin wheat gene as internal controls.

### 2.4. Cloning, Expression, and Production of Recombinant *Tt*ASR1 Protein in *E. coli*

The full-length open reading frame (ORF) of *Tt*ASR1 was amplified from the durum wheat variety Mahmoudi cDNA using two following primers corresponding to the 5′ and 3′ ends and containing *EcoR*I restriction sites at their ends. The *Tt*ASR1 ORF was cloned into the *EcoR*I site of the *Escherichia coli* expression vector pGEX-4T-1, resulting in a fusion with glutathione S-transferase (GST). The *Tt*ASR1 protein was expressed and purified as previously described [15].

### 2.5. *In Vivo* Assay of *E. coli* Stress Tolerance

Effects of heat and cold stress on the growth of *E. coli* strain harboring recombinant plasmid (*Tt*ASR1) or empty vector (GST) were studied. *E. coli* BL21 (DE3) cells were grown overnight at 37°C in LB containing ampicillin (100 *μ*g/ml). The primary culture was diluted 10-fold in LB containing antibiotics as secondary culture and was allowed to grow at 37°C. Isopropyl *β*-D-1-thiogalactopyranoside (IPTG) was added to culture when OD reached 0.6. The culture was induced for 2 h. Uninduced and induced cultures were diluted to 1 : 1000 and incubated at 4°C for 24 h or 50°C for 45 min; then 10 *μ*l of each sample was spread on LB agar plates with antibiotic and grown at 37°C overnight. Cell viability was estimated by counting the number of colony-forming units after incubation of the plate overnight at 37°C. The viability ratio of the transformations under heat and cold was calculated according to the following formula: viability ratio = (mean of colony number on a stressed plate/mean of colony number on control LB plate) × 100%.

For the kinetic assay, the bacterial suspension was grown at 37°C to OD = 0.3, and IPTG was added to a final concentration of 0.5 mM. Then, the culture was incubated again at 37°C. Every 1 h optical density *A*_600_ was measured.

### 2.6. Western Blot Analysis

For Western blot, recombinant *E. coli* BL21 strain was grown at 37°C to OD = 0.3, and IPTG was added to a final concentration of 0.5 mM and the culture was incubated again at 37°C. An appropriate volume (0.5 mL) of the culture was harvested by centrifugation every 1 h. Pellets were suspended in 100 *μ*L of protein sample loading buffer. The obtained samples were separated by 12% sodium dodecyl sulfate-polyacrylamide gel electrophoresis (SDS-PAGE) and transferred onto a polyvinylidene difluoride membrane (Millipore Corp). The membrane was blocked with milk and then probed with the GST-Tag antibody (sigma, 1/5000 dilution). After extensive washing in PBS (1X) and 0,01%Tween 20, the HRP-conjugated anti-rabbit IgG concentrate (Molecular Probes,1/10,000 dilution) was added as a secondary antibody. Signal detection was revealed using the ECL Western detection kit (Amersham Biosciences).

### 2.7. LDH Protective Assay

A solution of the freeze-labile lactate dehydrogenase (LDH) enzyme (EC1.1.1.27, rabbit muscle lactate dehydrogenase) from Sigma (Tokyo, Japan) was prepared (20 *μ*g/ml) in 10 mM sodium phosphate, pH 7.4. A mixture containing LDH was mixed with an equal volume of buffer containing 20 *μ*g/ml of bovine serum albumin (BSA) or TtASR1. Samples were then submitted to heat stress treatment. To determine the LDH activity, 20 *μ*l of LDH mixture was made up to 1 ml with the assay buffer (10 mM sodium phosphate, pH 7.4, 2 mM NADH, and 10 mM pyruvic acid).

NADH oxidation was monitored by measuring the absorbance A340 over 3 min, during which the reaction rate was linear. The rate of absorbance was then used to calculate activity ∆DO/min × 8095 = U/l (Biomaghreb kit). All samples were assayed in triplicate.

### 2.8. Stress Tolerance and Growth Assays of Yeast Cells

Two yeast strains were used in this study: the wild type KT1112 (MATa his3 leu2 ura3-52) [16] and the mutant KT1623 (KT1112 glc7-127), a weak salt sensitive and slow growth at 24–30°C [17]. The complete ORF of *Tt*ASR1 cDNA was cloned in the pYES2 expression vector (Invitrogen). This vector is a 2 *μ* based multicopy yeast plasmid and contains the URA3 gene and the Gal1 promoter for selection and expression in yeast. The recombinant pYES2-*Tt*ASR1 and the empty vector pYES2 were introduced into yeast cells using the PEG/LiAC transformation method as described [18]. The effects of TtASR1 expression on yeast cells in normal and stress conditions were studied on minimal medium (MM) (0.67% yeast nitrogen base, 40 mg/l tryptophan, 20 mg/l histidine, 120 mg/l leucine, and 40 mg/l adenine) supplemented with 2% glucose (MMGlc) or 2% galactose (MMGal) as carbon source. The recombinant yeast cells were grown overnight on MMGlc at 30°C. The serial dilutions of overnight yeast clone cultures were prepared (10^−1^ to 10^−4^). These dilution series were used to evaluate the sensitivity of yeast cells to various stress treatments. Each dilution was then spotted onto solid MMGal containing or not NaCl (1 M) and mannitol (0.5 M) and incubated at 30°C for three days.

### 2.9. Bioinformatics Analyses

Identification of the conserved domain of *Tt*ASR1 was carried out by query against the Conserved Domain Database (CDD) (http://www.ncbi.nlm.nih.gov/Structure/cdd/wrpsb.cgi) and BLAST program (http://blast.ncbi.nlm.nih.gov/Blast.cgi). Open reading frame and protein prediction were made using Softberry (http://linux1.softberry.com/berry.phtml?topic=bestorf&group=programs&subgroup=gfind). Some physicochemical properties were studied using the ExPASy server (http://web.expasy.org/protparam/). Hydrophobicity index was calculated using FoldIndex (http://bip.weizmann.ac.il/ Sequence alignment of the ASR1 proteins was performed using MUSCUL (http://www.ebi.ac.uk/Tools/services/msa/muscle/).

## 3. Results

### 3.1. *Tt*ASR1 Is a Novel ASR Gene from *Tritucum turgidum* L. subsp. *durum*

**Figure 1:**
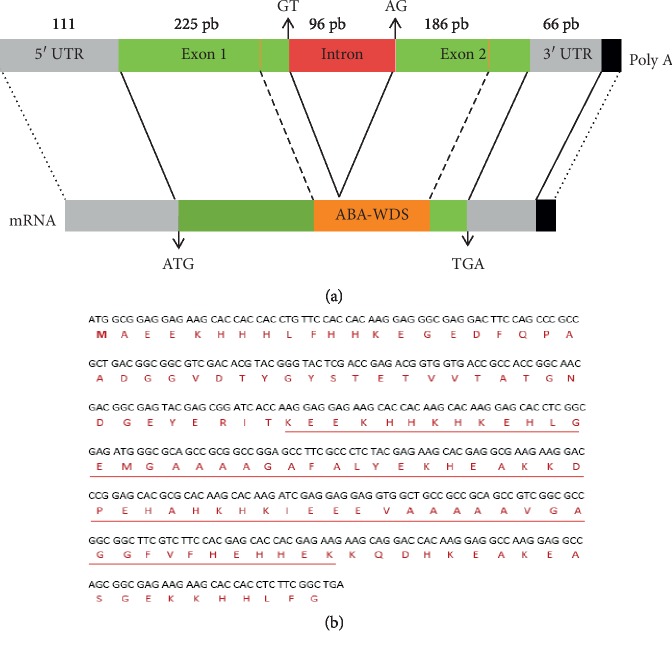
Isolation of TtASR1 from durum wheat. (a) Schematic representation of genomic and cDNA structure of TtASR1. Genomic DNA contains two exons that are separated by one intron. (b) The full-length cDNA sequence and the deduced amino acid sequence of TtASR1. The conserved domain ABA-WDS was marked by underline.

Abscisic acid, stress, and ripening family gene is widely distributed in higher plants. Using primers based on ASR cDNAs conserved region, a 0.5 kb DNA fragment from Mahmoudi was amplified, cloned, and sequenced. This sequence was used as a query to retrieve the contig (UCW_Tt-k45_contig_942) from the Graingene wheat transcriptome database and to design primers for full-length *Tt*ASR1 gene isolation. The PCR reaction was performed using Mahmoudi genomic DNA as a template and a 700 bp was obtained. The structure of the *Tt*ASR1 gene was predicted using plant-dedicated SofBerry software. The *Tt*ASR1 gene and cDNA sequences were deposited in GenBank under the accession numbers KX660742 and KX660744, respectively. The *Tt*ASR1 gene was about 684 bp long. It consists of the 5′-noncoding region (111 bp), two exons (E1 and E2 of 225 bp and 186 bp, respectively) interrupted with one intron (96 bp), and 3′-noncoding region of 66 bp (Figure 1(a)). The *Tt*ASR1 gene is located on chromosome 4AL (at positions 55, 656–56, 794). The open reading frame of *Tt*ASR1 is 411 bp long encoding a single peptide of 136 amino acid residues. The analysis of deduced *Tt*ASR1 protein sequence against InterPro and CDD revealed the presence of a characteristic abscisic acid-water-deficit stress (ABA_WDS) domain PFAM (PF02496) of the ASR family (Figure 1(b)).

**Figure 2:**
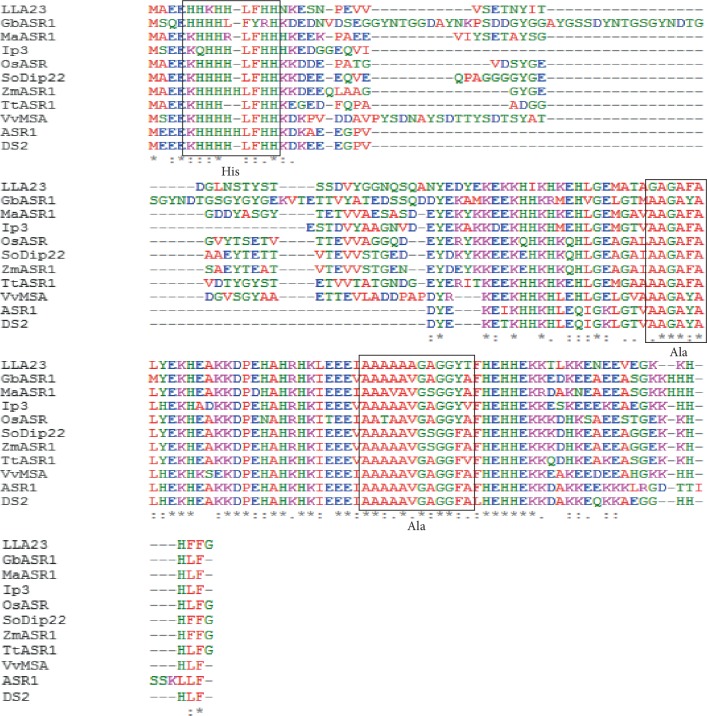
Amino acid sequence alignment of TtASR1 with other plant ASR proteins. Durum wheat TtASR1 was aligned with *Cucumis melo* ASR1, *Saccharum officinarum* SoDip22, *Oryza sativa* OsASR1, *Lilium longiflorum* LLA23, *Musa acuminate* MaASR, *Pinus taeda* Ip3, *Ginkgo biloba* GbASR, *Vitis vinifera* VvASR, *Solanum tuberosum* DS2, and *Zea mays* ZmASR1. Totally conserved residues were shaded in red. The alignment was done by Multalin. One His-rich region and two Ala-rich regions were underlined.

The deduced *Tt*ASR1 protein is a small protein, rich in Glu (16.1%), Ala (14.6%), His (13.9%), and Lys (12.4%) and no Cys and Trp residues (Supplementary [Supplementary-material supplementary-material-1]). Furthermore, the TtASR1 sequence carried 28 negatively charged (Asp + Glu) and 18 positively charged (Arg + Lys) residues (Supplementary [Supplementary-material supplementary-material-1]). The analysis of amino acid sequences of some ASR proteins from other species using ExPASy tools showed that although the amino acid sequences of ASR proteins were diverse in length, composition, and molecular weight, these proteins exhibit a low hydrophobicity ranging from 0.34 to 0.37 and they have a very close isoelectric point. *Tt*ASR1 shared maximum identity (67%) with *S. officinarum* and (66.42%) with *Z. mays* (Supplementary [Supplementary-material supplementary-material-1]). Multiple alignments of the putative *Tt*ASR1 amino acid sequence with ASRs from other plant species using MUSCLE showed two highly conserved regions: a small N-terminal consensus of 13 amino acids containing a rich His region and a long C-terminal region amino containing the ABA-WDS domain (PF02496) with 33 amino acids totally conserved. This region also exhibits two Ala-rich conserved regions (Figure 2).

### 3.2. Expression Pattern of *Tt*ASR1 under Abiotic Stress Conditions in Two Contrasting Tunisian Durum Wheat Landraces (*cv.* Mahmoudi and *cv.* Azizi) for Salt Tolerance

**Figure 3:**
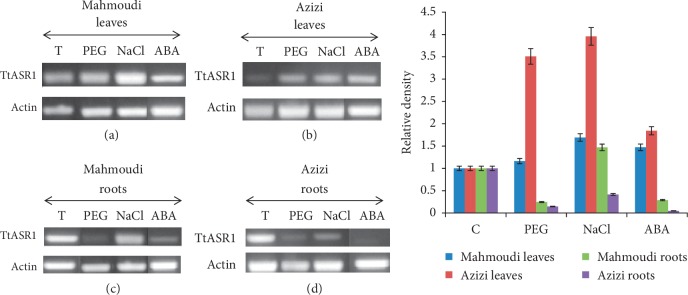
Expression of TtASR1 in response to abiotic stress and ABA. (a) RT-PCR showing the induction of TtASR1 in leaves of Mahmoudi variety following PEG, salt, and ABA treatments associated with densitometric analysis of the images of RT-PCR products. (b) RT-PCR showing the induction of TtASR1 in leaves of Azizi variety following PEG, salt, and ABA treatments associated with densitometric analysis of the images of RT-PCR products. (c) RT-PCR showing the induction of TtASR1 in roots of Mahmoudi variety following PEG, salt, and ABA treatments associated with densitometric analysis of the images of RT-PCR products. (d) RT-PCR showing the induction of TtASR1 in roots of Azizi variety following PEG, salt, and ABA treatments associated with densitometric analysis of the images of RT-PCR products. Densitometric data are presented as the ratio of pixel intensities for TtASR1 versus actin.

The expression pattern of *Tt*ASR1 in response to abiotic stress (PEG, NaCl, and ABA) in both leaves and roots was analyzed in the two Tunisian durum wheat landraces, Azizi and Mahmoudi, known to be sensitive and tolerant to salt, respectively. The different stress treatments were performed on 10-day-old seedlings for 24 h. Upon nonstress conditions, the TtASR1 gene was expressed in roots and leaves of the two studied durum wheat genotypes (Figure 3(a)–3(d)). Under NaCl, PEG, and ABA, *Tt*ASR1 expression was induced in leaves in both genotypes. In Azizi and Mahmoudi roots, *Tt*ASR1 expression was strongly repressed under PEG and ABA treatment (Figure 3(d)). Interestingly, the *Tt*ASR1 is specifically induced by salt only in Mahmoudi roots (the salt-tolerant genotype) (Figures 3(c) and 3(d)); in contrast, the *Tt*ASR1 expression was always repressed in Azizi roots (the salt-sensitive genotype) by salt (Figure 3(d)). The upregulation of the TtASR1 gene is specific to tolerant durum landraces roots (Mahmoudi) under salt stress, supporting the implication of this gene in the salt tolerance mechanism.

### 3.3. Overexpression of TtASR1 Improves *E. coli* Growth and Increases Its Resistance to Heat and Cold

**Figure 4:**
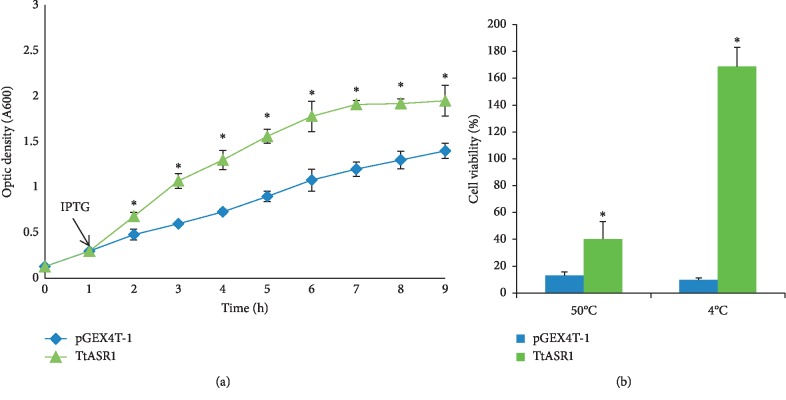
The growth performance of TtASR 1 cells. (a) Growth of IPTG induced *E. coli* cultures producing TtASR 1 or control pGEX4T-1 with a standard medium at 37°C. The increasing density of the liquid cultures was measured at 600 nm absorbance. (b) Cells viability ratio of *E. coli* transformed with pGEX4T-1-TtASR1 and pGEX4T-1 constructs under cold and heat stress. The values are the mean ± SE from three samples. Significant differences in the absorbance and cell viability are indicated as ^*∗*^*p* < 0.05 evaluated with Student's *t*-test.

To examine the potential role of *Tt*ASR1 in cellular responses under different temperatures conditions (37°C, 50°C, and 4°C), *Tt*ASR1 protein was heterologously expressed in *E. coli* (BL21). In standard condition, at 37°C, the cells expressing TtASR1 showed better growth than those with an empty vector (Figure 4(a)). To investigate the protective properties of TtASR1 *in vivo*, the cell viability of *E. coli* cells overexpressing TtASR1 or the empty vector was assessed under 50°C and 4°C. The overexpression of TtASR1 resulted in up to a 3-fold and 18-fold increase of CFUs under heat and cold stress treatments, respectively, in comparison to control strains expressing empty vector (Figure 4(b)). These data confirm that heterologous expression of TtASR1 protein in *E. coli* enhances its growth under standard conditions and its tolerance to heat and cold stress. The protein expression of TtASR1 in *E. coli* was analyzed by SDS-PAGE and by Western blot ([Supplementary-material supplementary-material-1]). Our results showed that this protein is expressed in *E. coli* at different times after induction by IPTG.

### 3.4. Effect of TtASR1 Protein on LDH Protective Activity under Heat Stress

**Figure 5:**
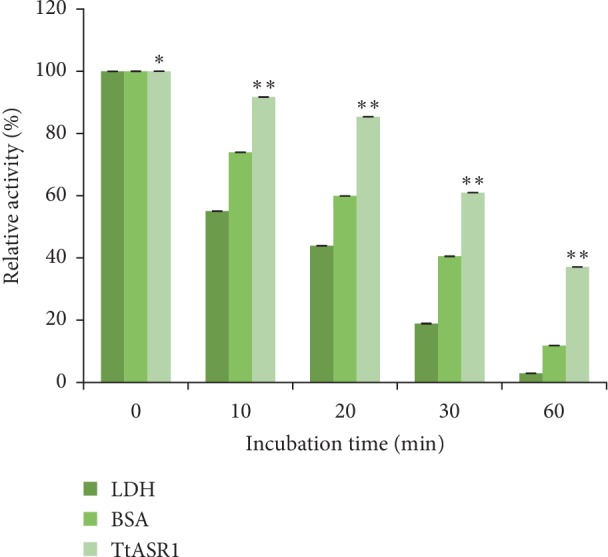
TtASR1 protein protects LDH activity from heat stress *in vitro*. LDH solutions were kept on heated at 43°C for the specified time in the absence or the presence of TtASR1 purified protein. A small aliquot was taken at each of several time points, and LD H activity was measured. Data are presented as relative activity (%) respective to the LDH activity registered before treatment. Three independent assays have been performed and standard errors are included. The values are the mean ± SE from three samples. Significant differences are indicated as ^*∗*^*p* < 0.05 and as ^*∗∗*^*p* < 0.001, evaluated with Student's *t*-test.

To evaluate the thermoprotective effect of *Tt*ASR1 *in vitro*, we have chosen the enzyme lactate dehydrogenase (LDH) as a substrate because it loses its activity under heat stress. The *Tt*ASR1 was purified and verified by sodium dodecyl sulfate polyacrylamide gel electrophoresis (SDS-PAGE). LDH was mixed with an equal volume of buffer containing BSA or TtASR1, and a time-dependent loss of activity was measured as described previously (see Materials and Methods section). LDH activity before treatment was regarded as 100%. As shown in Figure 5, without protectant (BSA) and *Tt*ASR1 protein, LDH activity was sharply decreased. In the first 10 min, LDH activity decreased by nearly 50% (Figure 5). During time and under heat stress, LDH loses gradually its activity, only 2% remaining after 60 min. In contrast, heat LDH inactivation was significantly reduced in the presence of *Tt*ASR1, with 40% of its initial activity being retained after 60 min (Figure 5). Although BSA showed protection after heat stress, *Tt*ASR1 protection is more pronounced. Altogether, our data showed that *Tt*ASR1 can protect LDH against heat stresses.

### 3.5. TtASR1 Confers Osmotic and Salt Stress Tolerance to Yeast Cells

To get further insight into the osmoprotective function of *Tt*ASR1, the effect of this protein was analyzed in yeast *S. cerevisiae*. Two yeast strains were used in this study, the wild type (KT1112) and a mutant one (KT1623), which is affected in the GLC7 gene encoding a PP1 (type 1 protein phosphatase-encoding gene) and exhibited salt-sensitive phenotype [17, 19]. The *Tt*ASR1 ORF was cloned in pYES2 yeast expression vector downstream of the inducible GAL1 promoter. The recombinant and the empty vectors, (pYES2-TtASR1) and (pYES2) respectively, were transferred to the two yeast strains (KT1112) and (KT1623).

**Figure 6:**
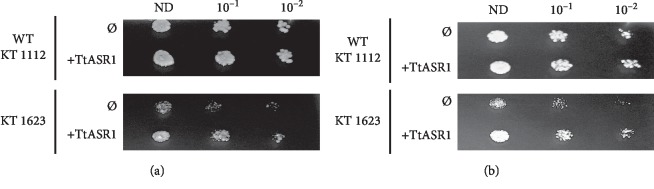
Expression of TtASR1 confers stress tolerance to yeast cells. Wild type and mutant yeast strains transformed with empty vector (Ø) or with TtASR1 (+TtASR1) were grown for 2 days under normal growth conditions (30°C) or under drought stress (mannitol 0.5 mM ) and salt stress NaCl 1 M) in minimum solid media containing galactose as carbon source. Shown growth tests are representative of at least three independent replicates. (a) NaCl 1 M. (b) mannitol 0.5 M.

After transformation, growth assays on solid media of yeast cells in the presence of galactose and under stress conditions were conducted. Under salt and mannitol, the wild type cells expressing *Tt*ASR1 grew slightly better than control cells (Figures 6(a) and 6(b)). However, the growth rate of mutant yeast cells (KT1623) expressing the *Tt*ASR1 on salt and mannitol was significantly better than control cells harboring empty vectors.

## 4. Discussion

Understanding the biochemical and molecular mechanisms for abiotic stress perception, transduction, and tolerance is still a major challenge. To cope with and adapt to different exogenous stimuli, plants have developed a variety of defense mechanisms. These include activation of a cascade of networks, mostly based on the manipulation of either transcription and/or signaling factors or genes that directly protect plant cells against stress [20]. ASR is a plant-specific gene family potentially involved in responses to drought, salinity stress [8, 21, 22], cold, and limited light [23]. On the other hand, the ASR gene from *Musa paradisiaca* was found upregulated by infection with *Fusarium oxysporum* [6]. The ASR genes family are not only involved in responses to abiotic and biotic stresses but also involved in processes of plant development, such as senescence, fruit ripening, and pollen maturation [23]. Back in 1993, the first ASR cDNA was screened from a tomato fruit (*Solanum lycopercicum*) cDNA library, by differential hybridization, with transcripts from tomato leaves under normal or water-stress conditions [7]. Soon thereafter, ASR genes have reported in many plant species other than tomato, ranging from ancient gymnosperms to monocots and dicots [21, 24–26]. However, there has been no report about the ASR in durum wheat (*Tritucum turgidum* L. subsp. *durum*), an economically and nutritionally important crop in the Mediterranean region. In our previous work, we showed experimentally that the TtASR1 is an intrinsically disordered protein (IDP) and undergoes structural transition under stress conditions (desiccation and heat) [15]. The present study describes the isolation and the partial functional characterization of the *Tt*ASR1, ASR gene from durum wheat (*Tritucum turgidum* L. subsp. *durum*). The *Tt*ASR1 gene, localized on the 4AL chromosome (at position s55, 656–56, 794), was 684 bp long and it contained a 411 pb open reading frame encoding a protein of 136 amino acids that belongs to the ABA-WDS (ABA-water-deficit stress Pfam PF02496) family.

TtASR1 is a small gene with two exons (225 bp and 186 bp) separated by one intron (96 bp). The simple TtASR1 structure is also shared by other species, for example, ASRs from *Solanum lycopercicum* [12], *Oryza sativa* [27], and *Salicornia brachiata* [28]. However, *Zm*ASR7-1, *Zm*ASR7-2, and *Zm*ASR7-3, ASRs from *Zea mays*, and *Sb*ASR6, *Sb*ASR7, ASRs from *Sorghum bicolor,* had their first and second exons fused [29].

The *Tt*ASR1 gene encoded a protein of 136 amino acid residues containing an N-terminal His-rich region and C-terminal conserved ABA-WDS domain (Pfam PF02496). The small N-terminal His-rich region is found in most ASRs proteins like Strawberry *Fa*ASR [30] and the soybean ASR protein (*Gm*ASR) [31]. The high His content was supposed to be involved preferentially in metal-binding [31]. In fact, in all glycophytic ASRs, the N-terminal consensus sequence has a stretch of six His residues in a 10 amino acid sequence that is typical for zinc-binding, while, in halophytic ASRs such as *Salicornia brachiata* and *Suaeda liaotungensis,* the N-terminal region is gly-rich region [32]. Similar to *Fa*ASR protein and SlASR protein (from *Suaeda liaotungensis*), the TtASR1 C-terminal region exhibited two Ala-rich rich regions [30, 32].

The *Tt*ASR1 expression pattern was studied in two Tunisian durum wheat landraces with contrasting behavior regarding salt tolerance, *cv.* Mahmoudi (salt-tolerant) and *cv.* Azizi (salt susceptible). The obtained results revealed that the TtASR1 transcript levels were responsive to different abiotic stress treatments at various degrees. Our data showed that *Tt*ASR1 was upregulated in the two durum wheat landraces leaves by different developmental (ABA) and environmental signals (PEG, salt).

Interestingly, TtASR1 expression in response to salt stress displayed distinctive patterns in the roots of the two local Tunisian durum genotypes. In Mahmoudi roots, TtASR1 was upregulated by salt stress, while it was downregulated in Azizi, supporting the implication of this gene in the salt tolerance mechanism. Similarly, the expression of the rice ASR5 gene was increased in the roots of the Al-tolerant rice cultivar Nipponbare; however, in the roots of the Al-sensitive rice cultivar Taim, ASR5 expression did not respond to aluminium (Al) treatment [33].

Cereals differ in their tolerance of salinity as we found that bread wheat (*Triticum aestivum*) is moderately tolerant and durum wheat (*Triticum turgidum ssp. durum*) is less tolerant [34]. Also, it is worthy of note that salinity affects plant growth by the osmotic stress of the salt around the roots, as well as by toxicity caused by excessive accumulation of salt in leaves. In this context, the *Tt*ASR1 gene may provide a potential functional marker to be useful for marker-assisted selection in a durum wheat breeding program for salt tolerance. The ABA, an important phytohormone, plays a critical role in response to various stress signals and controls the stomatal closure to prevent the intracellular water loss under drought conditions [2]. The *Tt*ASR1 was induced in the leaves of the two studied durum genotypes and repressed in their roots by ABA treatment. The ASR gene from *Ginkgo biloba* (*Gb*ASR) could be induced by ABA in roots and leaves [25]. The *Brachypodium distachyon Bd*ASR genes, except *Bd*ASR4, were found upregulated in leaves after ABA treatment [35]. In peach, the expression of *Pp*ASR could be induced in leaves by ABA [36]. In a context where no functional characterization has yet been carried out for the ASR gene from durum wheat, the TtASR1 was overexpressed in the yeast *Saccharomyces cerevisiae* and in *E. coli* to test its putative protective effects. Indeed, *E. coli* and yeast are being used as models for functional analysis of stress-responsive genes from plant origin [37]. In fact, yeast expression system was largely used to study the stress tolerance of some genes, for example, LEA4 gene from *Brassica napus* that enhanced the cellular tolerance to temperature and salt stresses of *E. coli* cells [38]. It was also reported that the expression of a protein gene from *Porteresia coarctata* (the serine-rich protein) conferred salt tolerance in yeast [39]. Heterologous expression of *Tt*ASR1 in yeast improved its growth under salt and osmotic stress. The KT1623, the yeast mutant strain, grows hardly under salt (NaCl, 1 M) and mannitol (mannitol, 0.5 M), but its resistance increased notably when overexpressing *Tt*ASR1. Similar results were found in ASR1 from rice [40] and the LEA gene from *Tamarix* [39]. The overexpression of *Tt*ASR1 improved *E. coli* cells growth under normal conditions (37°C) compared to control cells and enhanced its tolerance to high and low temperatures (4°C and 50°C). In the same context, the heterologous expression of *Mp*Asr in *E. coli* enhanced the tolerance of transformants to osmotic stress [6]. Recently, it was shown that the recombinant *Zn*ASR1 protein from wild jujube (*Ziziphus nummularia*) was proven to enhance drought stress tolerance in *E. coli* cells [37]. In line with the protective effect, our data showed that *Tt*ASR1 had protected the reporter enzyme (LDH) against heat denaturation *in vitro*. The LDH assay data support the hypothesis that ASR may function as a chaperone-like protein to confer heat tolerance by protecting some plant proteins from heat inactivation. The protective effect of LDH *in vitro* against heat stress has been described previously for ASR proteins from tomato [41], banana [6], and lily [42].

## 5. Conclusion

In our previous study, we showed that *Tt*ASR1 protein is intrinsically disordered proteins (IDP) and undergoes structural transitions under dehydration and heat or in the presence of zinc. In the present work, the corresponding gene was identified and characterized. Our results showed that *Tt*ASR1 protein could function as a chaperone-like protein and improve the viability of *E. coli* under heat stress and increase yeast tolerance under salt and osmotic stress. Interestingly, *Tt*ASR1 expression in response to salt stress displayed distinctive patterns in roots of the two local Tunisian durum genotypes with contrasting behavior regarding salt tolerance. Also of note is that the *Tt*ASR1 transcript was upregulated only in roots of the salt-tolerant Tunisian landraces (Mahmoudi) supporting the implication of this gene in the salt tolerance mechanisms. Taken together, our results confirm that *Tt*ASR1 plays an important role in durum wheat abiotic stress tolerance and may provide a potential functional marker to be useful for marker-assisted selection in a durum wheat breeding program for salt tolerance.

## Data Availability

No data were used to support this study.

## Abbreviations

abrABA: Abscisic acid
ABA-WDS: Abscisic acid-water-deficit stress domain
ASR: Abscisic acid, stress and ripening
BSA: Bovine serum albumin
LDH: Lactate dehydrogenase
LEA: Late embryogenesis abundant
IUPs: Intrinsically unstructured proteins
IPTG: Isopropyl *β*-D-1-thiogalactopyranoside
PEG: Polyethylene glycol.

## Supplementary Materials

Supplementary Materials Figure S1: analysis of TtASR1 protein patterns by SDS-PAGE and Western blot. (A) SDS-PAGE analysis of *E. coli* BL21 strain harboring recombinant plasmids encoding the TtASR1 protein. Lane M: molecular weight marker 97 to 14 kDa; lane 1: pellet fraction of uninduced BL21-pGEX-4T-1-TtASR1; pellet fraction of induced BL21-pGEX-4T-1-TtASR1; lane 2: 1h after induction; lane 3: 2 h after induction; lane 4: 3 h after induction; lane 5: 4 h after induction; lane 6: 5 h after induction; lane 7: 6 h after induction. The SDS-PAGE gel was stained with Coomassie Blue G-250. (B) Western blot analysis of *E. coli* BL21 strain harboring recombinant plasmids encoding the TtASR1 protein. Another identical SDS-PAGE gel (12%) with the same samples was used for protein transfer on a polyvinylidene difluoride membrane (Millipore Corp) and analyzed by Western blot using the GST-Tag antibody. Lane 1: pellet fraction of uninduced BL21- pGEX-4T-1-TtASR1; pellet fraction of induced BL21- pGEX-4T-1-TtASR1; lane 2: 1 h after induction; lane 3: 2 h after induction; lane 4: 3 h after induction; lane 5: 4 h after induction; lane 6: 5 h after induction; lane 7: 6 h after induction. Table S1: primers used for PCR amplification of the TtASR gene. Table S2: percentage of abundant amino acid of ASR gene from different plant species. Table S3: physiochemical properties analysis of TtASR1- and ASR-like proteins from different plant species using Expasy tools.
