# Supplementary material for: Abscisic Acid, Stress, and Ripening (TtASR1) Gene as a Functional Marker for Salt Tolerance in Durum Wheat
Source: Biomed Res Int. 2020 Jan 31;2020:7876357. doi: 10.1155/2020/7876357 (PMC7013306; doi:10.1155/2020/7876357)
Supplement: Supplementary Materials — Figure S1: analysis of TtASR1 protein patterns by SDS-PAGE and Western blot. (A) SDS-PAGE analysis of E. coli BL21 strain harboring recombinant plasmids encoding the TtASR1 protein. Lane M: molecular weight marker 97 to 14 kDa; lane 1: pellet fraction of uninduced BL21-pGEX-4T-1-TtASR1; pellet fraction of induced BL21-pGEX-4T-1-TtASR1; lane 2: 1h after induction; lane 3: 2 h after induction; lane 4: 3 h after induction; lane 5: 4 h after induction; lane 6: 5 h after induction; lane 7: 6 h after induction. The SDS-PAGE gel was stained with Coomassie Blue G-250. (B) Western blot analysis of E. coli BL21 strain harboring recombinant plasmids encoding the TtASR1 protein. Another identical SDS-PAGE gel (12%) with the same samples was used for protein transfer on a polyvinylidene difluoride membrane (Millipore Corp) and analyzed by Western blot using the GST-Tag antibody. Lane 1: pellet fraction of uninduced BL21- pGEX-4T-1-TtASR1; pellet fraction of induced BL21- pGEX-4T-1-TtASR1; lane 2: 1 h after induction; lane 3: 2 h after induction; lane 4: 3 h after induction; lane 5: 4 h after induction; lane 6: 5 h after induction; lane 7: 6 h after induction. Table S1: primers used for PCR amplification of the TtASR gene. Table S2: percentage of abundant amino acid of ASR gene from different plant species. Table S3: physiochemical properties analysis of TtASR1- and ASR-like proteins from different plant species using Expasy tools. [file 7876357.f1.docx]

**Figure S1:** Analysis ofTtASR1 protein patterns by SDS-PAGE and Western blot. **(A)** SDS-PAGE analysis of *E. coli* BL21 strain harboring recombinant plasmids encoding the TtASR1 protein. Lane M, Molecular weight marker 97 to 14 kDa; lane 1: pellet fraction of uninduced BL21- pGEX-4T-1-TtASR1; Pellet fraction of induced BL21- pGEX-4T-1-TtASR1 lane 2: 1h post-induction ; lane 3: 2 h post-induction; lane 4: 3 h post-induction; lane 5: 4 h post-induction; lane 6: 5 h post-induction; lane 7: 6 h post-induction. SDS-PAGE gel was stained with Coomassie Blue G-250. **(B)** Western blot analysis of *E. coli* BL21 strain harboring recombinant plasmids encoding the TtASR1 protein. Another identical SDS-PAGE gel (12%) with the same samples was used for protein transfer on a polyvinylidone difluoride membrane (Millipore Corp) and analyzed by Western blot using the GST-Tag antibody. Lane 1: pellet fraction of uninduced BL21- pGEX-4T-1-TtASR1; Pellet fraction of induced BL21- pGEX-4T-1-TtASR1 lane 2: 1h post-induction; lane 3: 2 h post-induction; lane 4: 3 h post-induction; lane 5: 4 h post-induction; lane 6: 5 h post-induction; lane 7: 6 h post-induction.

**Table S1:** Primers used for PCR amplification

| Primers | Sequences |
| --- | --- |
| P1 | 5’ATGGCGGAGGAGAAGCACCACCAC 3’ |
| P2 | 5’ TCAGCCGAAGAGGTGGTGCTTCTTC 3’ |
| P8 | 5’ CTGGGAACGGAACTGGTAGT 3’ |
| P9 | 5’ GAGAAGATGGGCCACAAAAG 3’ |
| Actin_Fw | 5’ GGCGATGAAGCTCAATCCAAACG 3’ |
| Actin_Rv | 5’ GGTCACGACCAGCAAGATCAAGACG 3’ |

**Table S2:** Percentage of abundant amino acid

| Proteins | [Alanine](https://fr.wiktionary.org/wiki/alanine)  (Ala)% | [Glutamate](https://fr.wiktionary.org/wiki/glutamate)  (Glu)% | [Histidine](https://fr.wiktionary.org/wiki/histidine)  (His)% | [Lysine](https://fr.wiktionary.org/wiki/lysine)  (Lys)% | [Cystéine](https://fr.wiktionary.org/wiki/cyst%C3%A9ine)  (Cys)% | [Tryptophane](https://fr.wiktionary.org/wiki/tryptophane) (Trp)% |
| --- | --- | --- | --- | --- | --- | --- |
| *TtASR1 (Triticum turgidum)* | 14.7 | 16.2 | 14 | 12.5 | 0 | 0 |
| *Lilium longflorum* | 11.3 | 15.5 | 12.7 | 12.0 | 0 | 0 |
| *Musa acuminate* | 14.7 | 17.5 | 14.7 | 12.6 | 0 | 0 |
| *Oryza sativa* | 13.0 | 15.2 | 13.0 | 13.0 | 0 | 0 |
| *Saccharum officinarum* | 14.1 | 19.7 | 13.4 | 13.4 | 0 | 0 |
| *Zea mays* | 15.2 | 19.6 | 14.5 | 13.0 | 0 | 0 |
| *Citrus maxima* | 13.3 | 17.3 | 12.2 | 13.3 | 0 | 0 |
| *Cucumis melo* | 12.5 | 19.6 | 21.4 | 13.4 | 0 | 0 |
| *Lycopersicon esculentum* | 13.0 | 15.7 | 15.7 | 17.4 | 0 | 0 |
| *Prunus armeniaca* | 8.5 | 14.0 | 11.0 | 8.5 | 0 | 0 |
| *Prunus persica* | 9.3 | 14.5 | 11.9 | 8.3 | 0 | 0 |
| *Solanum tuberosum* | 13.8 | 17.4 | 18.3 | 16.5 | 0 | 0 |
| *Vitis vinifera* | 14.8 | 14.1 | 14.8 | 10.7 | 0 | 0 |
| *Ginkgo biloba* | 10.5 | 13.3 | 10.5 | 8.8 | 0 | 0 |
| *Pinus taeda* | 12.7 | 18.3 | 16.7 | 13.5 | 0 | 0 |

**Table S3:** physiochemical properties analysis of TtASR1 and ASR like-proteins

| Proteins | % identity with ASR1Tt ^a^ | Lenght (aa) | Molecular weight (Da) | Theoretical  pI | Negatively charged residues  (Asp +Glu) | Positively charged residues  (Arg +Lys) | Hydrophobicity average ^b^ | GenBank or UniProt accession |
| --- | --- | --- | --- | --- | --- | --- | --- | --- |
| TtASR1 | - | 136 | 15127 | 5.99 | 28 | 18 | 0.377 | KX660742 |
| *Lilium longflorum* | 53.28 | 142 | 16108 | 6.11 | 26 | 18 | 0.364 | AAF15307 |
| *Musa acuminate* | 62.04 | 143 | 16263 | 5.99 | 31 | 20 | 0.362 | AAT35818 |
| *Oryza sativa* | 63.50 | 138 | 15464 | 6.20 | 27 | 20 | 0.362 | AAB96681 |
| *Saccharum officinarum* | 67.88 | 142 | 15932 | 5.78 | 33 | 20 | 0.355 | AAT57940 |
| *Zea mays* | 66.42 | 138 | 15542 | 5.89 | 31 | 19 | 0.360 | CAD12677.1 |
| *Citrus maxima* | 56.12 | 98 | 10872 | 6.03 | 21 | 15 | 0.363 | AAA82741 |
| *Cucumis melo* | 51.78 | 112 | 12901 | 6.22 | 25 | 16 | 0.353 | AAL27560 |
| *Solanum lycopersicum* | 56.52 | 115 | 13129 | 6.81 | 23 | 21 | 0.370 | AAB64185 |
| *Prunus armeniaca* | 57.66 | 200 | 21240 | 5.64 | 38 | 21 | 0.353 | AAB97140 |
| *Prunus persica* | 51.82 | 193 | 20759 | 5.68 | 38 | 21 | 0.352 | AAL26889 |
| *Solanum tuberosum* | 60.31 | 109 | 12428 | 6.45 | 23 | 18 | 0.351 | AAD00254 |
| *Vitis vinifera* | 62.38 | 149 | 16703 | 5.68 | 33 | 17 | 0.371 | AAZ93634 |
| *Ginkgo biloba* | 52.55 | 181 | 20111 | 5.33 | 38 | 19 | 0.346 | AAR23420 |
| *Pinus taeda* | 51.82 | 126 | 14376 | 5.99 | 29 | 18 | 0.355 | AAB02692 |

^a^Identity: (http://www.genome.jp/tools/clustalw/)

^b^ Phobicity index estimated using Foldindex : (http://bip.weizmann.ac.il/fldbin/findex)
